# Supplementary material for: In vitro ribosome synthesis and evolution through ribosome display
Source: Nat Commun. 2020 Feb 28;11:1108. doi: 10.1038/s41467-020-14705-2 (PMC7048773; doi:10.1038/s41467-020-14705-2)
Supplement: Supplementary file 1 — Supplementary Information [file 41467_2020_14705_MOESM1_ESM.pdf]

In vitro ribosome synthesis and evolution through ribosome display

Michael J. Hammerling[†1], Brian R. Fritz[†1], Danielle J. Yoesep[1], Do Soon Kim[1], Erik D. Carlson[1,6], and Michael C. Jewett[1,2,3,4,5,*]

[1] Department of Chemical and Biological Engineering,
[2] Center for Synthetic Biology
[3] Simpson Querrey Institute
[4] Chemistry of Life Processes Institute
[5] Robert H. Lurie Comprehensive Cancer Center
[6] Present address: Department of Chemical Engineering, Stanford University, Stanford, CA 94305, USA

Northwestern University, 2145 Sheridan Road, Evanston, IL 60208, USA.

* To whom correspondence should be addressed. Tel: +1 847 467 5007; Email: m-jewett@northwestern.edu

[†] These authors contributed equally.

Keywords: synthetic biology, ribosome construction, *in vitro*, directed evolution

Submitted to *Nature Communications*

Supplementary Information

Supplementary Tables

Construct	Template plasmid	Backbone plasmid	Assembly method	Forward primer (5'-3')	Reverse primer (5'-3')
pRDV-sfGFP	pY71sfGFP	pRDV	Digestion-Ligation	GGTGGT**CCATGG**GCAGCAAAGGTGAAGAACTGTTTAC	GGTGGT**AAGCTT**CGTAATACCTGCCGCATTC
pRDV-HH (1 of 2)	p23S-HH	-	Gibson assembly	GCCATTGGACTTCAAACCGCCCTTAGGTCTGAGCGTGATACC	CTTTGTTAGCAGCCGGATCTCAAAAAACCCCTCAAGACCC
pRDV-HH (2 of 2)	pRDV	-	Gibson assembly	GGGTCTTGAGGGGTTTTTTGAGATCCGGCTGCTAACAAAG	GGTATCACGCTCAGACCTAAGGGCGGTTTGAAGTCCAATGGC
pT7rrnB-NF (16S - C967Δ)	pT7rrnB	-	Phos-Ligation	/Phos/AACGCGAAGAACCTTACCTG	/Phos/CATCGAATTAAACCACATGCTCC
pT7rrnB-NF (23S - G2251A)	pT7rrnB	-	Phos-Ligation	/Phos/AGGCGGTCTCCTCCTAAAG	/Phos/CAGTCAAACTACCCACCAGAC
pRDV-FLAG	pRDV	-	Phos-Ligation	/Phos/GACTACAAAGACGATGACGACAAGCTTTATATGGCCTCGGG	/Phos/CATGGATATATCTCCTTCTTAAAGTTAAAC
pRDV-3xFLAG	pRDV	-	Phos-Ligation	/Phos/GATCATGATATCGATTACAAGGATGACGATGACAAGCTTTATATGGCCTCGGG	/Phos/TTTATAATCACCGTCATGGTCTTTGTAGTCCATGGATATATCTCCTTCTTAAAGTTAAAC
pRDV-His	pRDV	-	Phos-Ligation	/Phos/GGCCATCATCATCATCATCATAAGCTTTATATGGCCTCGGG	/Phos/CATGGATATATCTCCTTCTTAAAGTTAAAC
pRDV-Strep	pRDV	-	Phos-Ligation	/Phos/GGCTGGAGCCACCCGCAGTTCGAAAAAAAGCTTTATATGGCCTCGGG	/Phos/CATGGATATATCTCCTTCTTAAAGTTAAAC
pRDV-GST	pGEX6p1	pRDV	Digestion-Ligation	GGTGGT**CCATGG**GTTCCCCTATACTAGGTTATTGGAAAATTA	GGTGGT**AAGCTT**CCCCTGGAACAGAACTTCC
pT7rrnBΔ660	pT7rrnB	-	Phos-Ligation	/Phos/GGTGGTGCTCTTCGTGAGACAGTTCGGTCCCTATCTG	/Phos/GGTGGTGCTCTTCAGGAATTTCGCTACCTTAGGACCG
pT7rrnB-Lib6E	pT7rrnB	-	Phos-Ligation	/Phos/CATATCGACGGCGGTGTTTGGCANCTCGNNGTCGGCTCATCACATCCTG	/Phos/AACTCTTGGGCGGTATCNGCCTGTTNTCCNCGGAGTACCTTTTATCCGTTG
pT7rrnB-Lib12NC	pT7rrnB	-	Phos-Ligation	/Phos/CANANCGNCGGNGNNGTTTGGCACCTCGATGTC	/Phos/NACNCTNGGGNGNNATCAGCCTGTTATCCCCG
pT7rrnB-LibCR	pT7rrnB	-	Phos-Ligation	/Phos/NNNNNNCCCCGTGAACCTTTACTATAGC	/Phos/CGTCTTGCCGCGGGT

Supplementary Table 1. Primers for plasmid constructions. Bold letters indicate cut sites used for digestion and ligation, where an insert was generated from PCR with the indicated primers, and the resulting PCR product and backbone plasmid were digested with the same restriction enzymes prior to ligation. Gibson assembly was performed according to literature (Gibson *et al.*, 2009). '/Phos/' indicates the use 5' phosphorylation of primers or PCR product with the enzyme polynucleotide kinase (PNK) prior to ligation.

Use	Forward primer (5'-3')	Reverse primer (5'-3')
23S rRNA RT-qPCR	CTACGGTGCTGAAGCAACAA	CGAAGTTACGGCACCATTTT
23S RT-PCR for 660 bp recovery	GGTGGT**GCTCTTC**TTCCTTGTCGGGTAAGTTCCGAC	GGTGGT**GCTCTTC**CTCACGACGTTCTAAACCCAGCTC

Supplementary Table 2. Primers for RT-qPCR and RT-PCR of 23S rRNA. Bold letters indicate cut sites used to reassemble operon from recovered and digested 23S cDNA and digested pT7rrnBΔ660.

Specificity using various wash buffers		Tween-20 (%)			
		**0.05**	**0.25**	**1.00**	**5.00**
**NaCl (M)**	**0.15**	120	150	126	119
	**0.30**	112	113	99	90
	**0.50**	106	95	113	86
	**1.00**	95	91	79	84

Supplementary Table 3. Optimization of NaCl and Tween-20 concentration in wash buffer for RISE method with 3xFLAG-tag. Values represent average specific capture of functional ribosomes relative to nonfunctional ribosomes as determined by RT-qPCR for two independent pairs of reactions.

23S position:	2461	2462	2464	2468	2471	2474	2477	2479	2482	2486	2488	2489	Other mutations
WT	A	C	G	A	A	U	U	U	A	C	G	U	
12NC-1	**G**	**A**	G	A	A	U	U	U	A	C	G	U	G2445C
12NC-2	A	**A**	**C**	A	A	**G**	**C**	U	A	**G**	**A**	U	
12NC-3	**U**	**G**	**C**	**G**	A	**G**	**C**	U	A	**G**	**U**	**A**	
12NC-4	**C**	**A**	**C**	**C**	**U**	**C**	**G**	**A**	A	**G**	**U**	**A**	A2101G
12NC-5	**C**	C	**A**	**G**	A	**C**	**A**	U	**U**	**U**	G	**G**	U1993C
12NC-6	**U**	C	G	A	A	U	U	U	A	C	G	U	G1968U, C2258A, C2306A

Supplementary Table 4. Isolated sequences from evolution of twelve non-conserved bases of the PTC of 23S rRNA after two RISE cycles. 23S rRNA genes for each selected library member were sequenced using Sanger sequencing. Mutations occurring at other bases within the recovered fragments of 23S rRNA are noted.

Genotype	p-value	BH
AUGAGC	0.000274	0.000781
AUGAGU	2.39E-09	4.78E-08
CGGAGA	0.9273	1
AUUAGA	0.7203	0.900375
AGCAGA	0.9221	1
CGGAGC	0.1299	0.185571
AUGAGA	0.02415	0.043909
AGAAGA	0.6387	0.8516
AGGAGC	1.73E-08	8.65E-08
GCGAGA	0.9873	1
AUUAGC	0.009706	0.024265
AUUAGU	5.89E-09	5.89E-08
AUAAGA	0.03524	0.058733
GUGAGA	0.01715	0.038111
GCGAGC	0.06227	0.0958
AUCAGA	6.57E-05	0.000219
UGAAGA	2.47E-05	9.88E-05
AGGAGA	9.32E-09	6.21E-08
AGCAGG	0.0213	0.0426
CGGAGU	1	1

Supplementary Table 5. Comparisons of sfGFP production in iSAT using evolved clindamycin-resistance genotypes versus clindamycin-resistant control. Production of sfGFP in iSAT was assessed in across 7 independent replicate reactions in the presence of 500 µM clindamycin and compared using a one-sided Welch's t-test to the clindamycin-resistant control 23S mutant A2058U to assess which mutants had greater activity than this control. These p-values were corrected using the Benjamini & Hochberg correction for multiple comparisons, and these values are reported in the "BH" column. Values in the BH column were determined to be significant if they were < 0.05.

Supplementary Figures

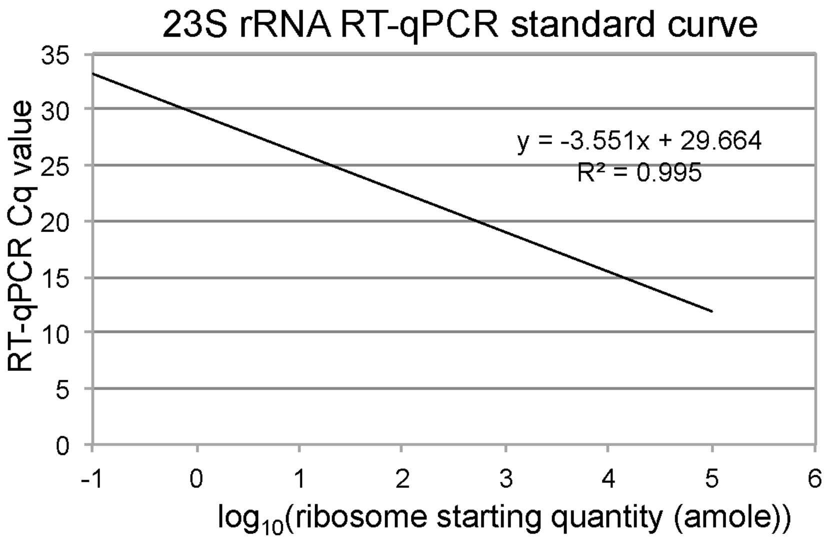

Supplementary Figure 1. 23S rRNA RT-qPCR standard curve for dilution series of purified 70S *E. coli* ribosomes. Purified 70S ribosomes were serially diluted 10-fold in nuclease-free water and used as templates in RT-qPCR.

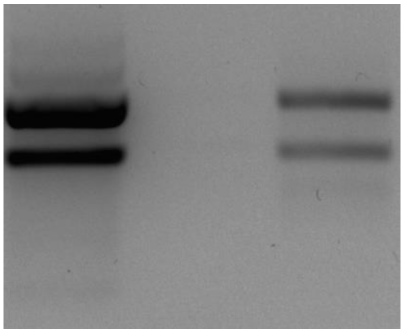

Supplementary Figure 2. Ribosome capture by ribosome display. Agarose gel shows ribosomes captured from iSAT reactions with nonfunctional (NF) or wild-type (WT) rDNA operon incubated for 1.5 h at 37°C. 4.5 pmol purified 70S ribosomes were run for comparison to represent the maximum theoretical number of iSAT ribosomes for 300 nM ribosomal proteins in a 15 μL reaction. Top band represents 23S rRNA and bottom band represents 16S rRNA. Gel representative of n=3 independent experiments.

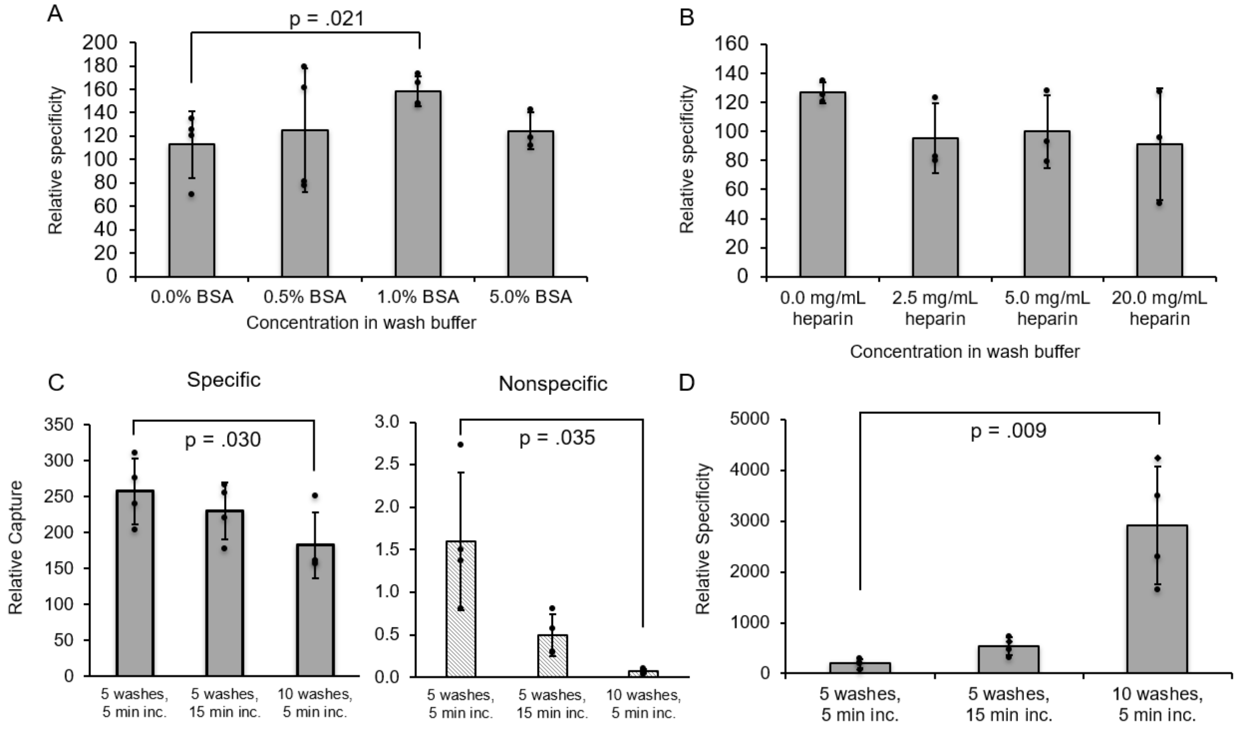

Supplementary Figure 3. Optimization of binding and wash buffers for RISE using 3xFLAG-tag. For the binding buffer, additives **(A)** BSA (% w/v) and **(B)** heparin were tested. For wash conditions, the number of washes and incubation of each wash were varied: **(C)** relative capture of iSAT ribosomes from specific capture (functional ribosomes) and non-specific capture (nonfunctional ribosomes), and **(D)** relative specificity of each wash condition. Values represent average specific capture of functional ribosomes relative to nonfunctional ribosomes as determined by RT-qPCR for at least three independent pairs of reactions. All tests of significance were one-sided Welch's t-tests. Values represent averages of four independent reactions (n=4) in A, C, and D and three independent reactions (n=3) in B, and error bars represent one standard deviation.

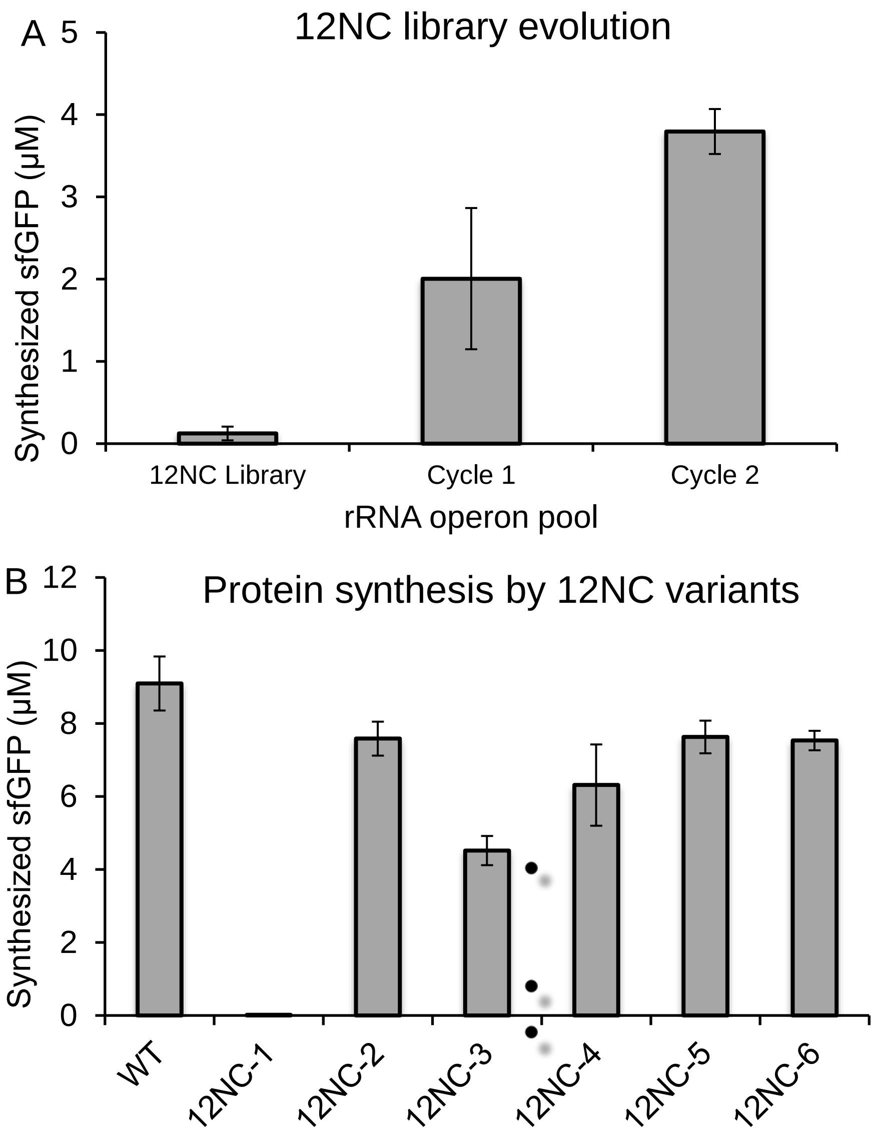

Supplementary Figure 4. *In vitro* evolution of 23S rRNA mutations in the peptidyl transferase center of *E. coli* ribosomes from RISE. (A) iSAT activity as measured by sfGFP production from rDNA operon pools recovered during PTC evolution by RISE. RISE was performed starting from rDNA operon libraries with degenerate mutation of twelve non-conserved bases of the PTC (12NC library) and rDNA pools were tested for iSAT activity. Values represent averages of at least three independent reactions and error bars represent one standard deviation. (B) iSAT activity as measured by sfGFP production of individual rDNA operon variants recovered from 12NC evolution using RISE. Variants were recovered and isolated after two RISE cycles. Variant sequences are listed in Supplementary Table S4. Values represent the average of three independent reactions (n=3) and error bars represent one standard deviation.

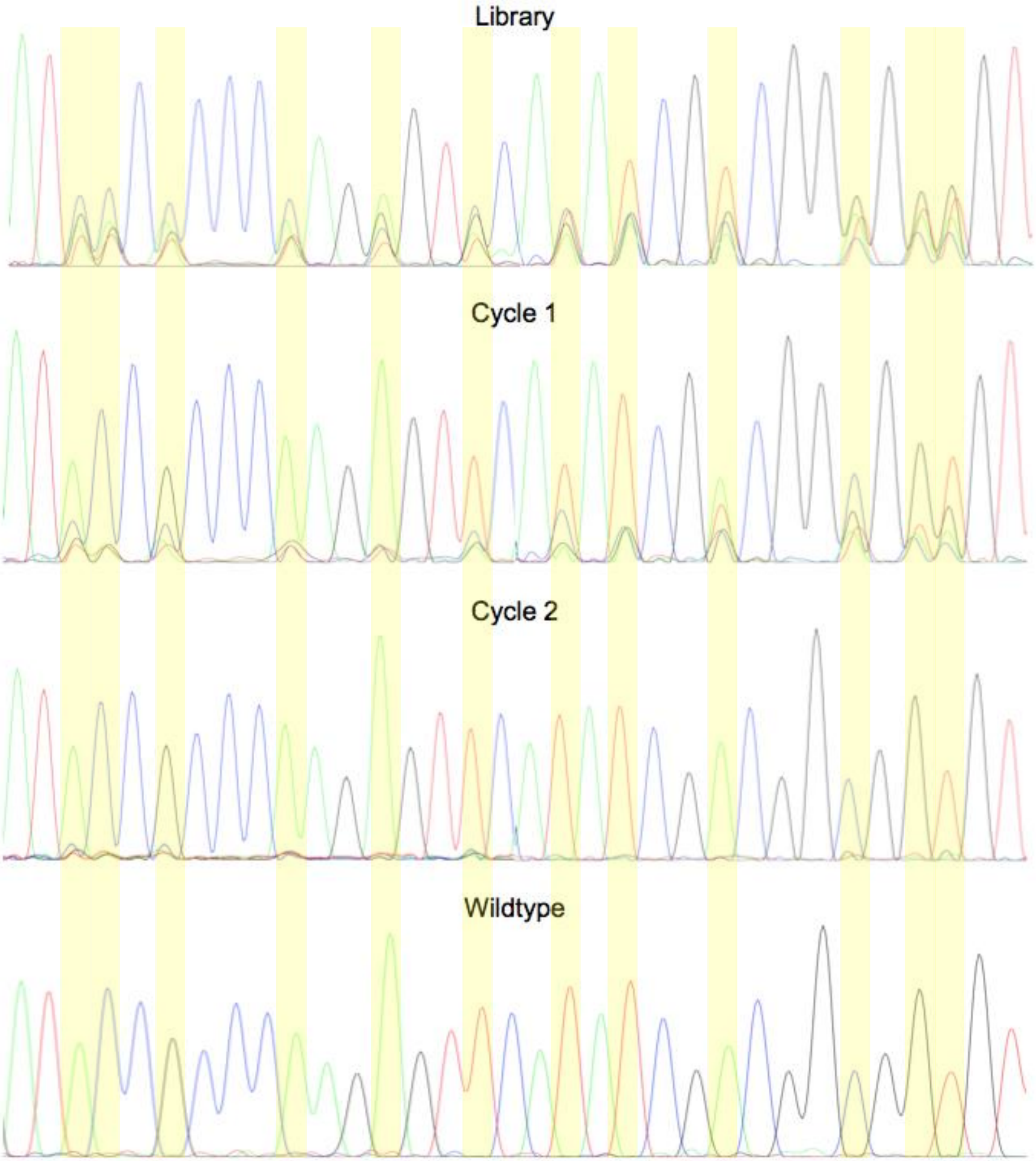

Supplementary Figure 5. Progression of rDNA operon sequence composition for evolution of 12 non-conserved 23S rRNA bases using RISE. Traces show bases 2459 to 2491 of 23S rDNA. Yellow is used to mark the 12 mutated bases. Black represents guanine, blue represents cytosine, green represents adenosine, and red represents thymine.

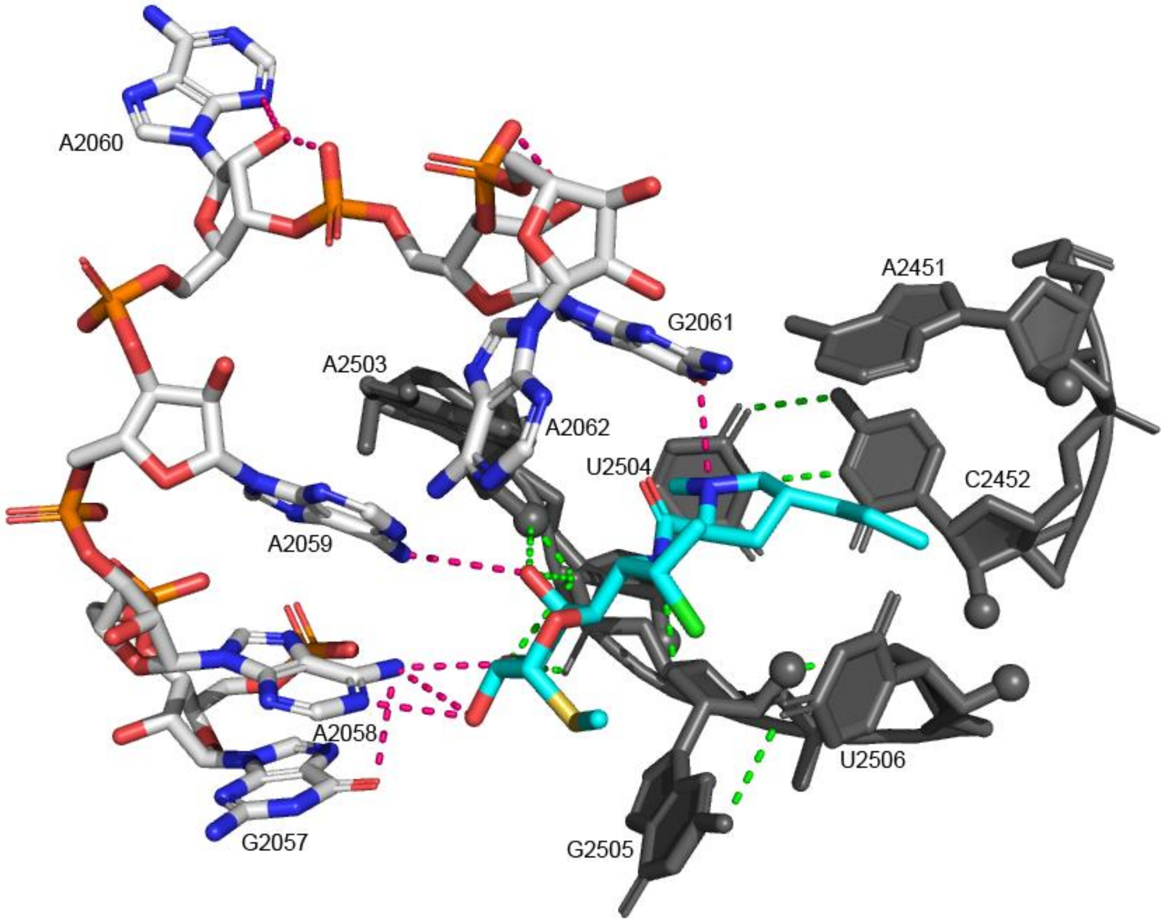

Supplementary Figure 6. Binding of residues of the 23S rRNA to clindamycin. Residues 2057-2062 (white sticks) comprising our clindamycin resistance library form up to five putative binding interactions (pink dashes) with clindamycin (cyan sticks). Other 23S rRNA positions within 5 angstroms of clindamycin (gray sticks) also form five putative interactions with clindamycin (green dashes). This structure was visualized using Pymol. PDB: 4V7V

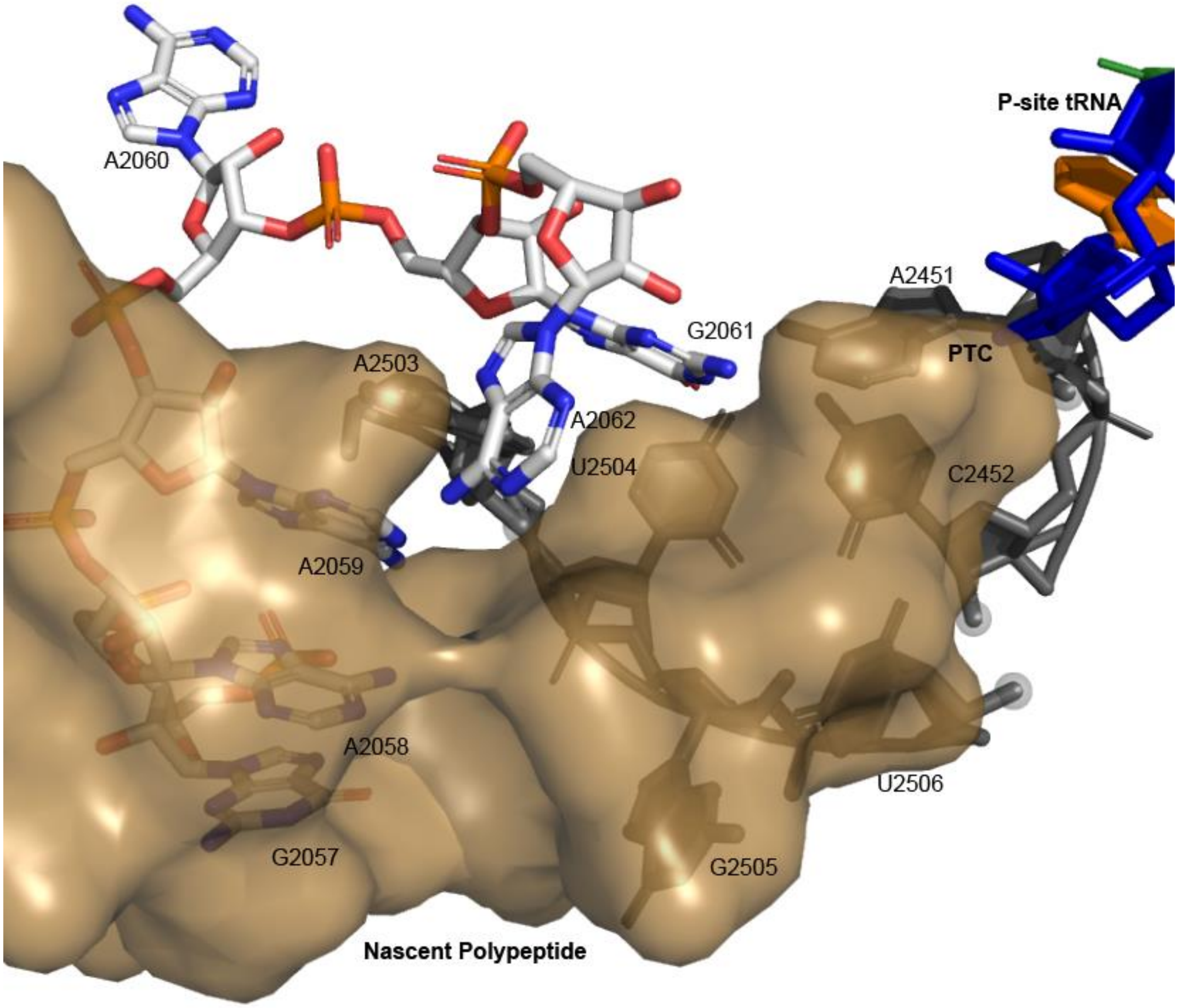

Supplementary Figure 7. Residues participating in clindamycin binding with the P-site tRNA and nascent polypetide. The P-site tRNA (blue sticks) and nascent polypeptide (beige surface) are depicted in the same orientation as Supplementary Figure S6. Positions 2057-2062 (white sticks) of clindamycin-resistance library extend away from the PTC toward the exit tunnel, limiting their participation in peptide bond formation. In contrast, other residues which interact with clindamycin (gray sticks) surround the nascent polypeptide and the PTC, limiting the possibility of mutating them to achieve clindamycin resistance while maintaining peptidyl-transferase activity. This structure was visualized using Pymol. PDB: 5NWY

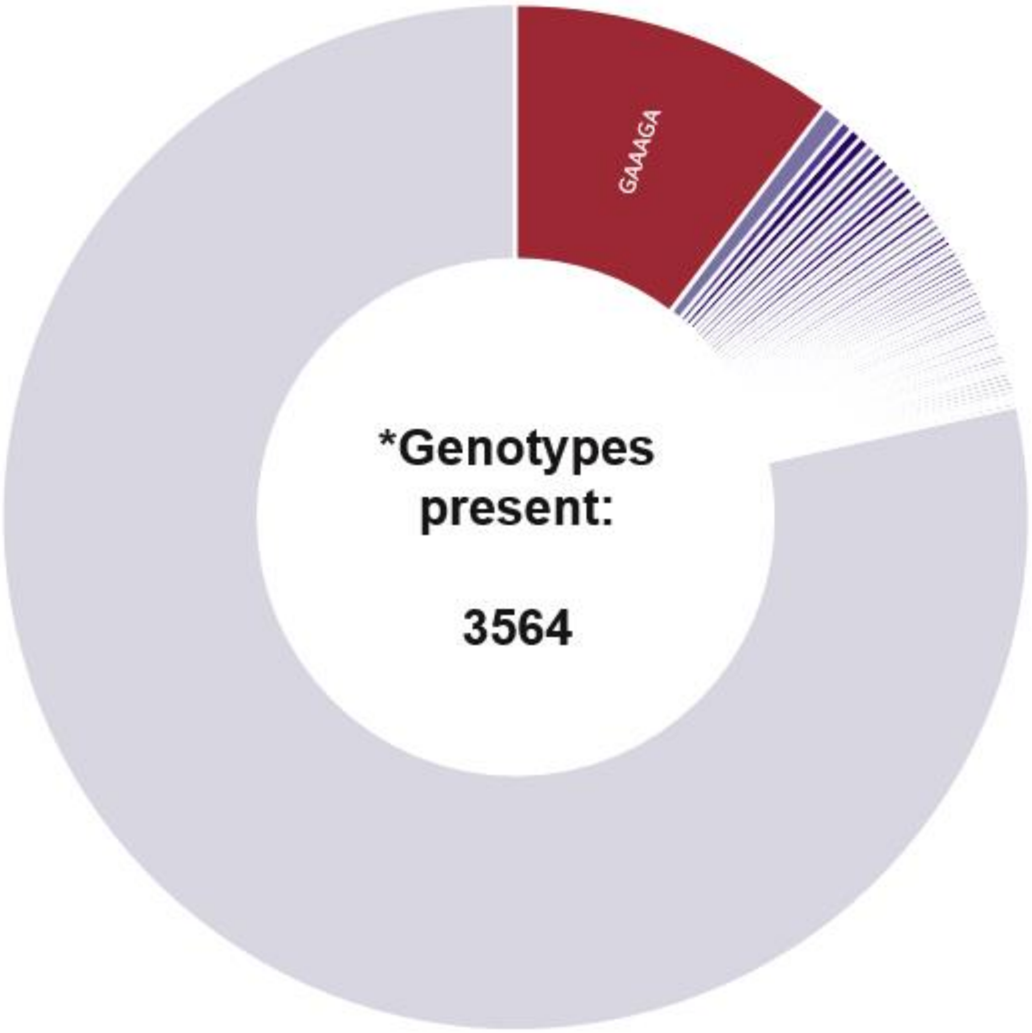

Supplementary Figure 8. Initial genotype diversity in degenerate 6N CR library. Sunburst plot depicting the initial genotype diversity in the CR library. Each slice represents the proportion of the initial population made up by the given genotype. The large gray slice represents all the genotypes at too low a frequency to depict graphically with an individual slice pooled into one group. The maroon slice indicates wild-type, revealing that the initial library was biased toward wild-type, likely due to incomplete cleavage of the wild-type plasmid when the library was built.

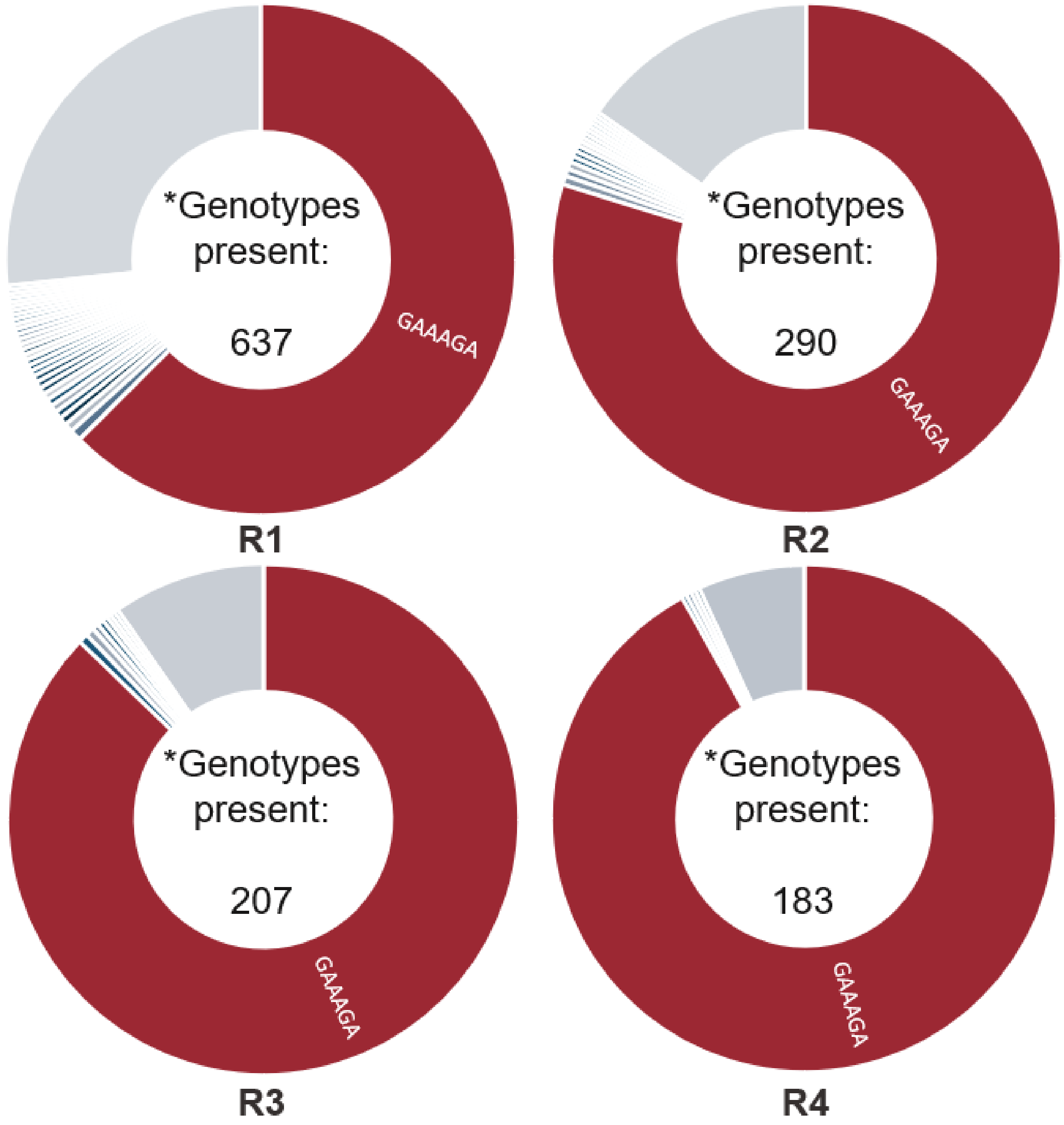

Supplementary Figure 9. Genotype diversity across four rounds of selection on the CR library in 0 µM clindamycin. While other genotypes are also seen increasing in frequency throughout the course of the selection, wild-type rapidly dominates the population over four rounds of selection in the absence of clindamycin. R'X' = Round X (e.g., 1, 2, 3, and 4). As expected, the wild-type sequence GAAAGA dominates the library after 4 rounds of selection.

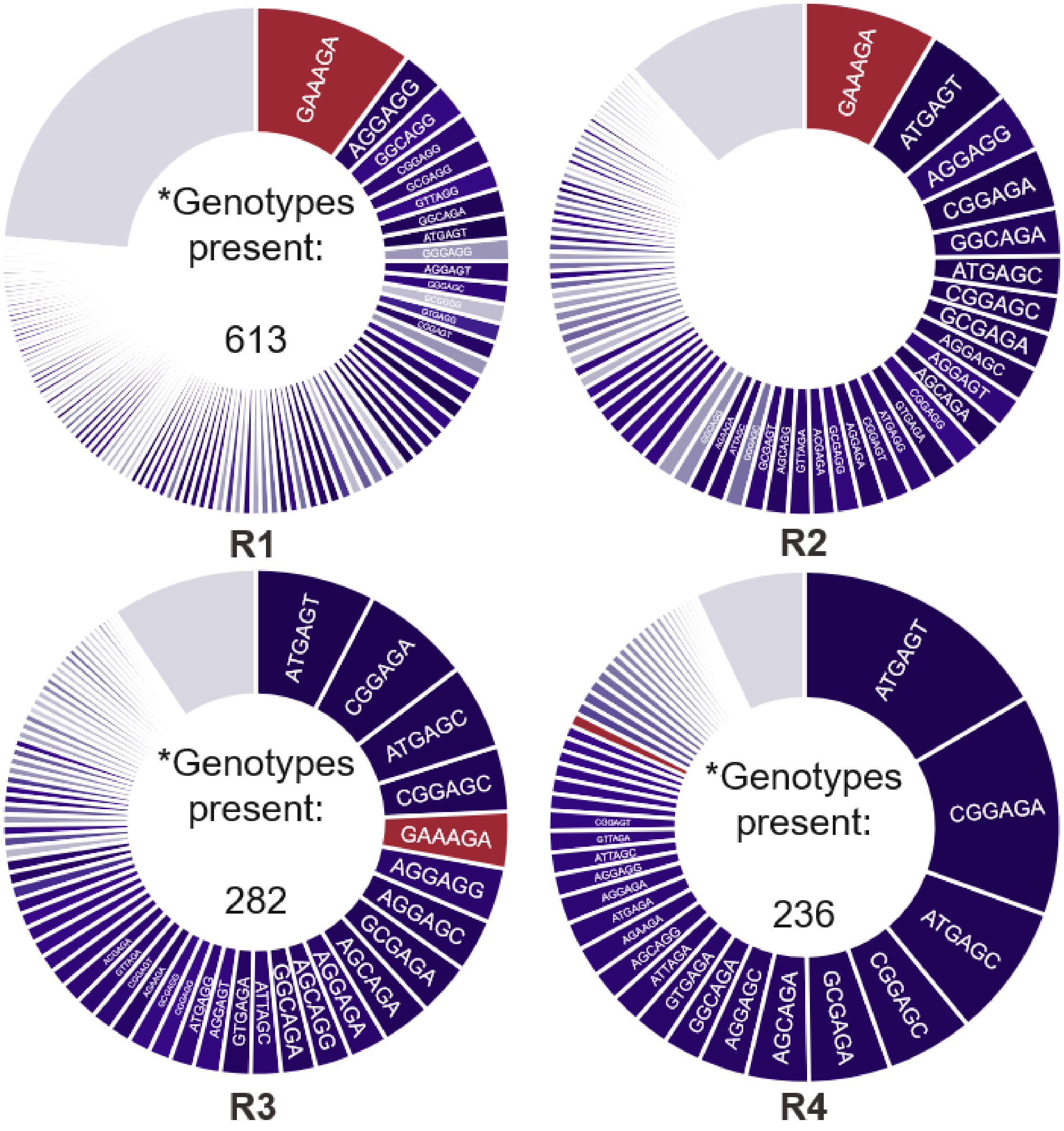

Supplementary Figure 10. Genotype diversity across four rounds of selection on the CR library in 500 µM clindamycin. Though wild-type starts out at high frequency, it is rapidly displaced by many clindamycin-resistant genotypes, indicating that our selection platform is specific for successfully translating ribosomes and is capable of selecting against wild-type under conditions where it does not translate efficiently. Shade of purple depicts ranking of frequency at the end of the selection. R'X' = Round X (e.g., 1, 2, 3, and 4).

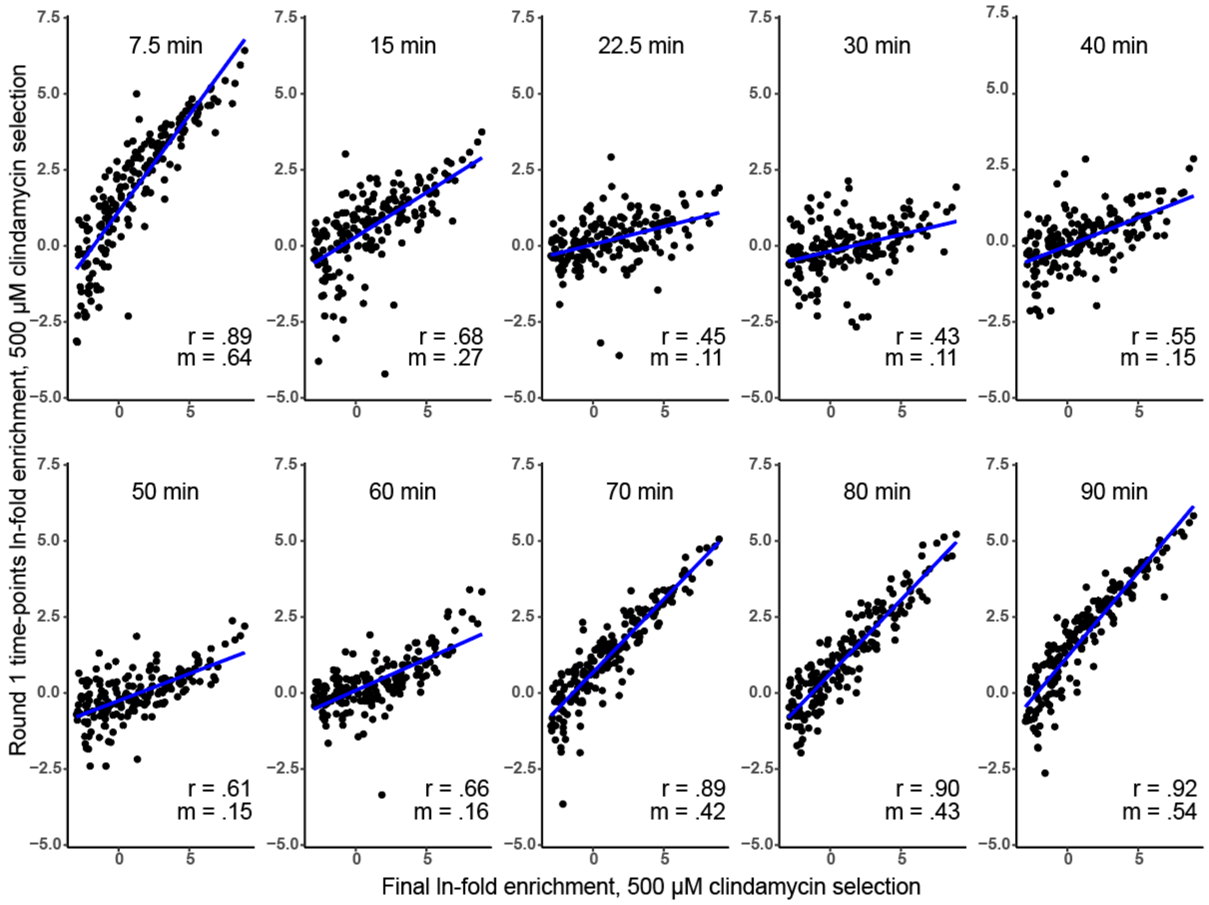

Supplementary Figure 11. Time course of CR genotype enrichment from degenerate library in 500 µM clindamycin. Each point represents a genotype whose ln-fold enrichment after four rounds of selection (x-axis) is plotted against its enrichment when the first round of selection is harvested at time points from 7.5 to 90 minutes. Since the most-enriched genotypes from the last round of selection are known to be highly active and clindamycin-resistant, the slope (m) and Pearson's correlation coefficient (r) are measures of the strength and fidelity of the selection for active CR ribosomes at each time point, respectively. As expected, ribosome display reactions harvested at 70-90 minutes show efficient selection, in addition to the somewhat more surprising outcome of efficient selection for active ribosomes at the 7.5-minute time point.

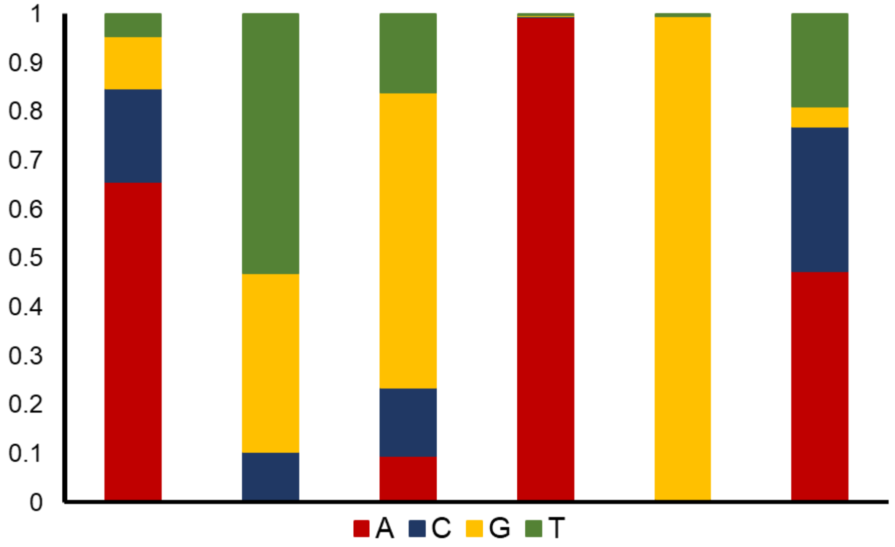

	2057	2058	2059	2060	2061	2062
A	65.6%	0.5%	9.4%	99.3%	0.3%	47.2%
C	19.2%	9.8%	14.0%	0.2%	0.3%	29.6%
G	10.6%	36.6%	60.5%	0.2%	98.9%	4.2%
T	4.7%	53.1%	16.1%	0.3%	0.5%	19.0%

ATGAGA

Consensus genotype

Expected	9.8%
Actual	3.2%
Fold-difference	**.34**

GCGAGC

Expected	.18%
Actual	1.4%
Fold-difference	**7.9**

TGAAGA

Expected	.07%
Actual	1.3%
Fold-difference	**18.0**

Supplementary Figure 12. Final nucleotide frequencies after four rounds of selection on 500 μM clindamycin. While two positions (2060, 2061) have nearly converged after four rounds of selection, the remaining four show biases but retain significant diversity. The consensus genotype after four rounds is ATGAGA, though this genotype is present at approximately 3-fold lower concentration than expected by simply multiplying the frequency of each constituent base of the genotype together. Two other genotypes, GCGAGC and TGAAGA, however, are present at 7.9- and 18.0-fold higher frequency than expected. These genotypes would likely never be discovered by studying the effect of mutations at single positions on activity and clindamycin-resistance, highlighting the power of an evolutionary approach for discovering new ribosome variants.

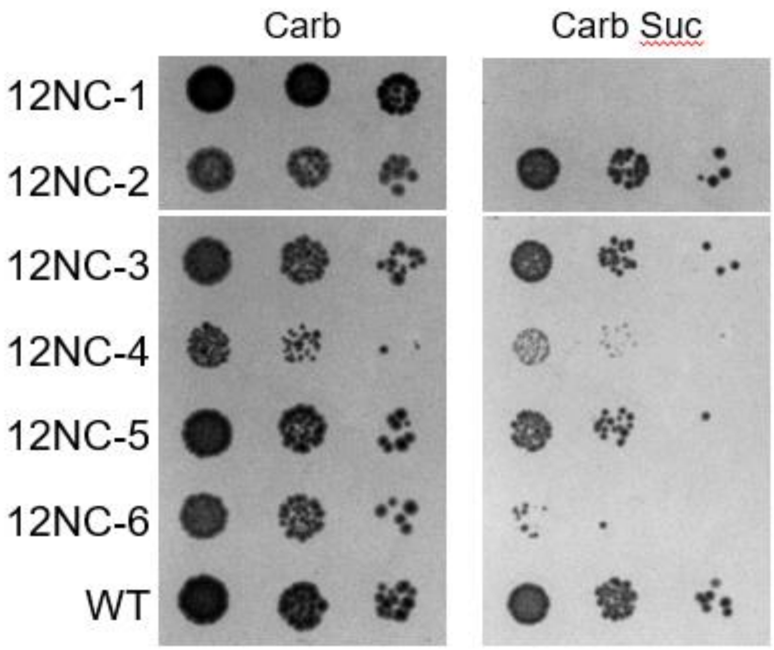

Supplementary Figure 13. Viability of 12NC ribosome variants *in vivo*. Isolated rDNA variants from evolution of the 12NC libraries were altered to include a native promoter for *in vivo* expression. Variants were transformed into the Squires strain and cells were plated by dilution series on (left) LB with 50 µg/mL carbenicillin and (right) LB with 50 µg/mL carbenicillin and 5% w/v sucrose. Plasmid constructs able to support cell growth on LB with 50 µg/mL carbenicillin and 5% w/v sucrose were purified and sequence-confirmed. Images are representative of at least 3 independent biological replicates.

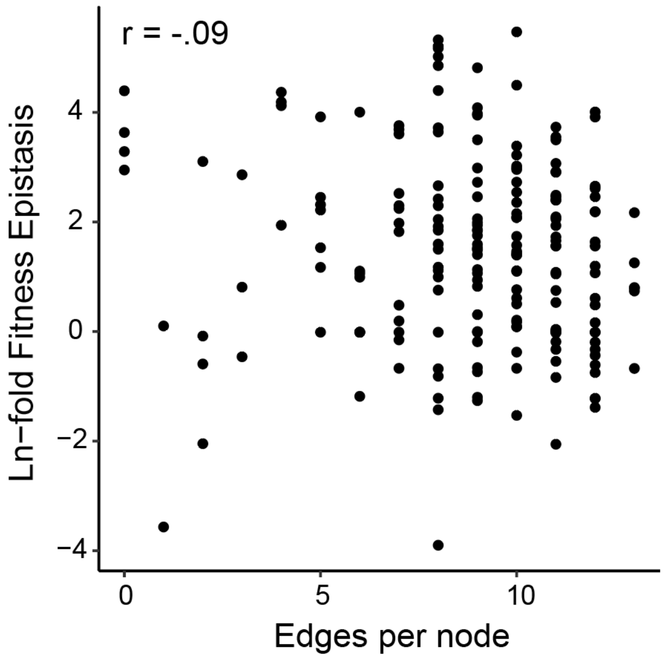

Supplementary Figure 14. Epistasis values of clindamycin resistant genotypes plotted against edges per node. Quantitative analysis of fitness and epistasis of the network of top clindamycin resistant genotypes shows that edges per node is not correlated with epistasis values (Pearson's r = -.09).

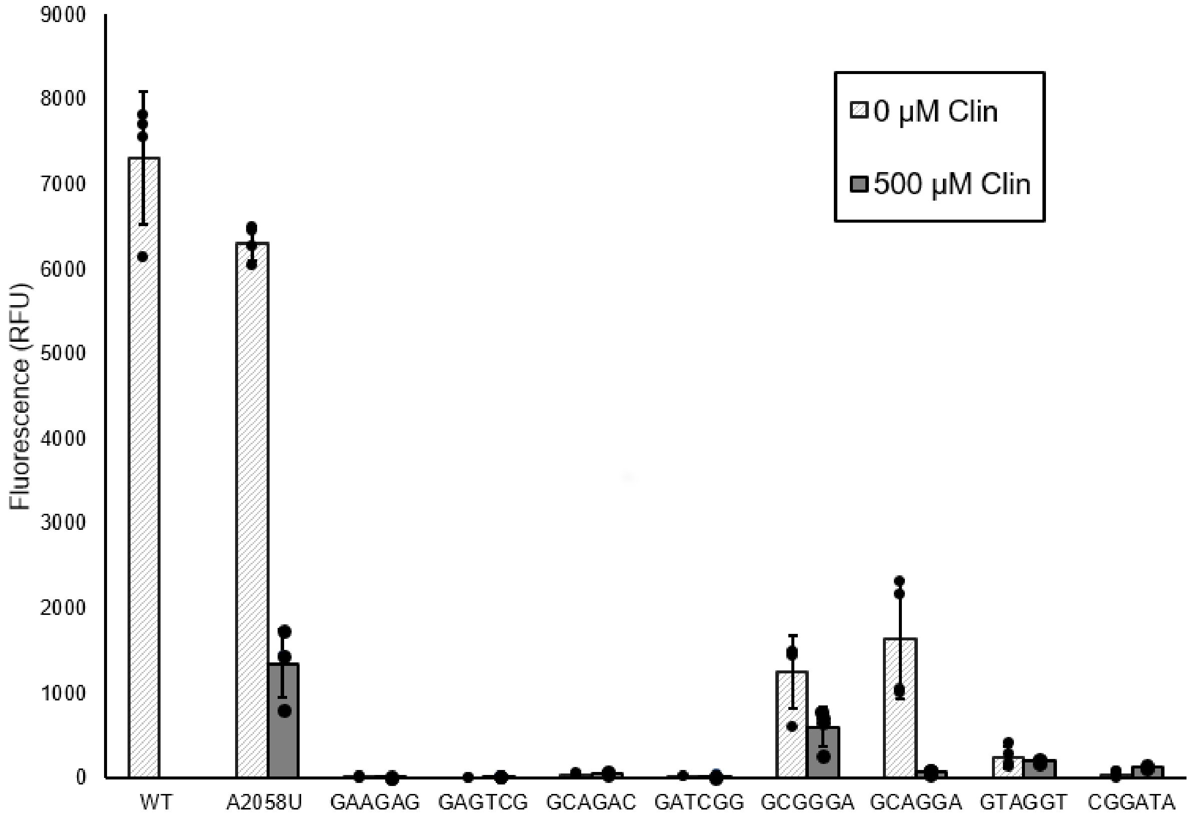

Supplementary Figure 15. Function of extreme positive epistasis mutants in iSAT. While several genotypes displayed extreme positive epistasis values in our evolution experiment, the question remained whether these mutants retained any translation activity in iSAT or if their presence at the end of the selection was an artifact of selection. This is especially true for mutants differing from wild-type at positions 2060 and 2061, which are generally thought to be essential for ribosome function. A number of such mutants were cloned and tested for sfGFP production in iSAT in 0 µM (striped bars) or 500 µM (gray bars) clindamycin. Several of these mutants (*e.g.,* GCGGGA, GCAGGA) retained measurable activity, indicating that they were indeed functional ribosomal mutants. Values represent mean values for four independent pairs of reactions (n=4). Error bars represent one standard deviation.
