# Supplementary material for: In vitro ribosome synthesis and evolution through ribosome display
Source: Nat Commun. 2020 Feb 28;11:1108. doi: 10.1038/s41467-020-14705-2 (PMC7048773; doi:10.1038/s41467-020-14705-2)
Supplement: Supplementary file 2 — Reporting Summary [file 41467_2020_14705_MOESM2_ESM.pdf]

nature research

Corresponding author(s): *Double-blind peer review submissions: write DBPR and your manuscript number here instead of author names.*

Last updated by author(s): *YYYY-MM-DD*

Reporting Summary

Nature Research wishes to improve the reproducibility of the work that we publish. This form provides structure for consistency and transparency in reporting. For further information on Nature Research policies, see Authors & Referees and the Editorial Policy Checklist.

Statistics

For all statistical analyses, confirm that the following items are present in the figure legend, table legend, main text, or Methods section.

n/a	Confirmed	
☐	☒	The exact sample size (*n*) for each experimental group/condition, given as a discrete number and unit of measurement
☐	☒	A statement on whether measurements were taken from distinct samples or whether the same sample was measured repeatedly
☐	☒	The statistical test(s) used AND whether they are one- or two-sided<br>*Only common tests should be described solely by name; describe more complex techniques in the Methods section.*
☒	☐	A description of all covariates tested
☐	☒	A description of any assumptions or corrections, such as tests of normality and adjustment for multiple comparisons
☐	☒	A full description of the statistical parameters including central tendency (e.g. means) or other basic estimates (e.g. regression coefficient) AND variation (e.g. standard deviation) or associated estimates of uncertainty (e.g. confidence intervals)
☐	☒	For null hypothesis testing, the test statistic (e.g. F, t, r) with confidence intervals, effect sizes, degrees of freedom and P value noted<br>*Give P values as exact values whenever suitable.*
☒	☐	For Bayesian analysis, information on the choice of priors and Markov chain Monte Carlo settings
☒	☐	For hierarchical and complex designs, identification of the appropriate level for tests and full reporting of outcomes
☐	☒	Estimates of effect sizes (e.g. Cohen's *d*, Pearson's *r*), indicating how they were calculated

Our web collection on statistics for biologists contains articles on many of the points above.

Software and code

Policy information about availability of computer code

Data collection	Commercial software used includes: Image Lab version 6.0.1 build 34 (Bio-rad Laboratories, Inc.), and CFX96™ Real-Time PCR Detection System (Bio-Rad)
Data analysis	Software for data analysis includes: Geneious Version 6.1.8, sickle version 1.33 (https://github.com/najoshi/sickle), Python version 2.7.15, and RStudio version 1.1.453. Pymol was used for molecular modeling.

For manuscripts utilizing custom algorithms or software that are central to the research but not yet described in published literature, software must be made available to editors/reviewers. We strongly encourage code deposition in a community repository (e.g. GitHub). See the Nature Research guidelines for submitting code & software for further information.

Data

Policy information about availability of data

All manuscripts must include a data availability statement. This statement should provide the following information, where applicable:

- Accession codes, unique identifiers, or web links for publicly available datasets
- A list of figures that have associated raw data
- A description of any restrictions on data availability

Next-generation sequencing data supporting this document are available at http://www.ncbi.nlm.nih.gov/bioproject/602158. Additional data that support the findings of this study are available from the corresponding author upon reasonable request.

Field-specific reporting

Please select the one below that is the best fit for your research. If you are not sure, read the appropriate sections before making your selection.

☒ Life sciences ☐ Behavioural & social sciences ☐ Ecological, evolutionary & environmental sciences

For a reference copy of the document with all sections, see nature.com/documents/nr-reporting-summary-flat.pdf

Life sciences study design

All studies must disclose on these points even when the disclosure is negative.

Sample size	Quantification of protein production from n=3 CFPS reactions were used to enable calculation of mean and standard deviation as is regular practice in cell-free literature. Quantification of growth rate for ribosomal RNA mutants were also performed with n=3 replicates. For enrichment and epistasis analysis of genotypes, the number of genotypes included depended on those which fit criteria denoted in Materials and Methods for analysis. This varied from hundreds to thousands of genotypes for each analysis.
Data exclusions	For deep sequencing analysis, genotypes were considered to be absent from the population if the number of reads observing that genotype were 5 or fewer. Excluding observations at the bottom of the detection limit is common for deep sequencing and for enrichment analysis, as the error rate of deep sequencing becomes problematic, and the noise of such analysis becomes amplified near the bottom of the detection limit.
Replication	All attempts at replication were successful.
Randomization	Samples were organized by experimental variables then characterized fully and reported in completion. Therefore, samples were not randomized.
Blinding	Blinding was not relevant. Animal or human participants were not used in this study.

Reporting for specific materials, systems and methods

We require information from authors about some types of materials, experimental systems and methods used in many studies. Here, indicate whether each material, system or method listed is relevant to your study. If you are not sure if a list item applies to your research, read the appropriate section before selecting a response.

Materials & experimental systems

n/a	Involved in the study
☒	☐ Antibodies
☒	☐ Eukaryotic cell lines
☒	☐ Palaeontology
☒	☐ Animals and other organisms
☒	☐ Human research participants
☒	☐ Clinical data

Methods

n/a	Involved in the study
☒	☐ ChIP-seq
☒	☐ Flow cytometry
☒	☐ MRI-based neuroimaging
